# Unintended spread of a biosafety level 2 recombinant retrovirus

**DOI:** 10.1186/1742-4690-6-86

**Published:** 2009-09-22

**Authors:** Alexander Stang, Elisabeth Petrasch-Parwez, Sabine Brandt, Rolf Dermietzel, Helmut E Meyer, Kai Stühler, Sven-T Liffers, Klaus Überla, Thomas Grunwald

**Affiliations:** 1 Department of Molecular and Medical Virology, Ruhr-University Bochum, D-44780 Bochum, Germany; 2 Department of Neuroanatomy and Molecular Brain Research, Ruhr-University Bochum, D-44780 Bochum, Germany; 3 Medical Proteome Center, Ruhr-University Bochum, D-44780 Bochum, Germany

## Abstract

**Background:**

Contamination of vertebrate cell lines with animal retroviruses has been documented repeatedly before. Although such viral contaminants can be easily identified with high sensitivity by PCR, it is impossible to screen for all potential contaminants. Therefore, we explored two novel methods to identify viral contaminations in cell lines without prior knowledge of the kind of contaminant.

**Results:**

The first hint for the presence of contaminating retroviruses in one of our cell lines was obtained by electron microscopy of exosome-like vesicles released from the supernatants of transfected 293T cells. Random amplification of particle associated RNAs (PAN-PCR) from supernatant of contaminated 293T cells and sequencing of the amplicons revealed several nucleotide sequences showing highest similarity to either murine leukemia virus (MuLV) or squirrel monkey retrovirus (SMRV). Subsequent mass spectrometry analysis confirmed our findings, since we could identify several peptide sequences originating from monkey and murine retroviral proteins. Quantitative PCRs were established for both viruses to test currently cultured cell lines as well as liquid nitrogen frozen cell stocks. Gene fragments for both viruses could be detected in a broad range of permissive cell lines from multiple species. Furthermore, experimental infections of cells negative for these viruses showed that both viruses replicate rapidly to high loads. We decided to further analyze the genomic sequence of the MuLV-like contaminant virus. Surprisingly it was neither identical to MuLV nor to the novel xenotropic MuLV related retrovirus (XMRV) but showed 99% identity to a synthetic retrovirus which was engineered in the 1980s.

**Conclusion:**

The high degree of nucleotide identity suggests unintended spread of a biosafety level 2 recombinant virus, which could also affect the risk assessment of gene-modified organisms released from contaminated cell cultures. The study further indicates that both mass spectrometry and PAN-PCR are powerful methods to identify viral contaminations in cell lines without prior knowledge of the kind of contaminant. Both methods might be useful tools for testing cell lines before using them for critical purposes.

## Findings

**Figure 1:**
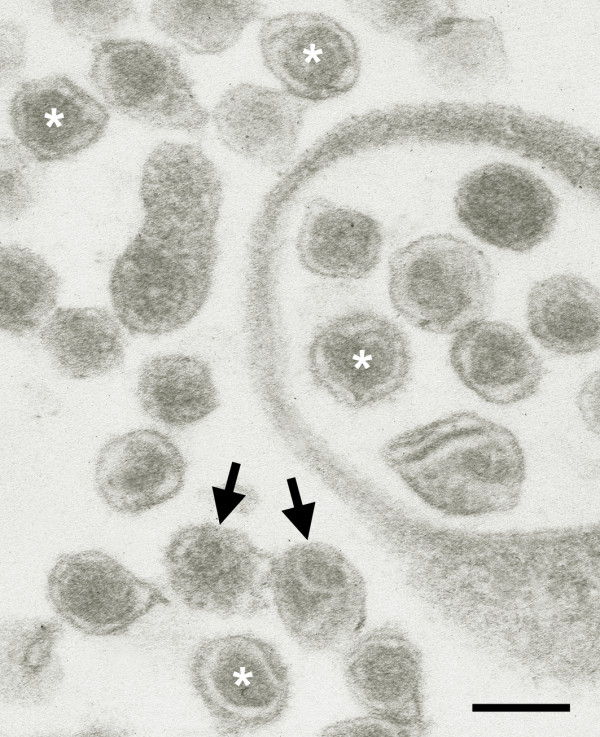
**Electron microscopical analysis of viral contaminants**. Viral particles from the supernatant of 293T cells were pelleted through a 20% sucrose cushion. Ultrathin sections of the fixed pellet show two types of particles resembling retroviruses. The more abundant one (*) exhibits a central electron-dense core, while the core of the other type of particles (→) is located excentrically. Scale bar: 100 nm.

The first evidence for a retroviral contamination was obtained by electron microscopy, originally performed to characterize the production of exosome-like vesicles released from transfected 293T cells. Vesicles were purified from supernatants by ultracentrifugation through a 20% sucrose cushion. Resulting pellets were fixed with 2,5% glutaraldehyde and 1% paraformaldehyde in 0.1 M sodium phosphate (pH: 7.4), postfixed with 2% osmium tetroxide, dehydrated and embedded in araldite (Serva). Ultrathin sections (100 nm) were contrasted with uranyl acetate and lead citrate, viewed in a Philips EM 420 electron microscope and documented by the digital system DITABIS (Digital Biomedical Imaging System). Surprisingly, in addition to the expected exosome-like vesicles the supernatants displayed two main types of enveloped viruses each of which with a diameter of around 100 nm (Fig. 1). One type exhibits a centrally located spherical electron-dense core closely resembling the type-C morphology of retroviruses that are shown by both murine leukemia viruses (MLV) and squirrel monkey retrovirus [1]. The other type which was much less frequent also shows a spherical outer shape, but displays an excentrically located core (Fig. 1). Of note, the latter type does not resemble the characteristic morphology of the two retroviruses identified below. Both types of particles were present in supernatants from both transfected and untransfected cells indicating that they were produced independently of proteins expressed by the transfected plasmids.

For identification of the unknown viruses a method based on the random amplification of particle associated RNAs (PAN-PCR) [2] was used. Isolation of nucleic acids from viral particles was done as described before [2] using 30 ml of supernatant from 293 T cells. Pellets were resuspended in 0.5 ml PBS and used for purification of viral RNA by means of the DNA Blood Mini Kit (Qiagen). For RNA preparation residual DNA was degraded by an RNase-free DNase (Ambion) and purified RNA was subsequently reverse transcribed to double stranded cDNA using the cDNA Synthesis Kit (Roche). Thereafter DNA and RNA (double stranded cDNA) were further processed equally.

Our previously published protocol of PAN-PCR [2] was slightly modified by including a random amplification protocol described elsewhere [3,4]. In detail, double stranded cDNA was digested with MseI and ethanol-precipitated in the presence of 1 μg glycogen. Adapter ligation was carried out in a total volume of 10 μl containing the MseI digested DNA, 400 U T4 DNA-Ligase and T4 DNA-Ligase buffer (New England Biolabs) and 20 pmol adapter composed by the hybridized oligonucleotides NBam24 (AGGCAACTGTGCTATCCGAGGGAG) and NCsp11 (TACTCCCTCGG) for 1 h at 4°C followed by 6 h at 16°C. 2 μl of this reaction were used for PCR amplification in a total volume of 25 μl containing 0.2 mM each dNTP, 10 mM Tris-HCl (pH 9.0), 1.5 mM MgCl_2_, 50 mM KCl, 1.25 U of Taq DNA Polymerase (Amersham Biosciences) and the primer NBam24 (1 mM). Two-step thermocycling was done by 30 cycles 95°C for 30 s and 72°C for 2 min followed by 10 min at 72°C. Products were used for cloning into the pCRII Vector as described by the manufacturer (Invitrogen). At least 40 colonies were picked and tested directly by PCR as described [2]. 25 PCR products of different size were sequenced and analyzed for homologies to viral sequences by a nucleotide-nucleotide (BLASTn) and translated BLAST search (BLASTx) at the NCBI website [5].

**Figure 2:**
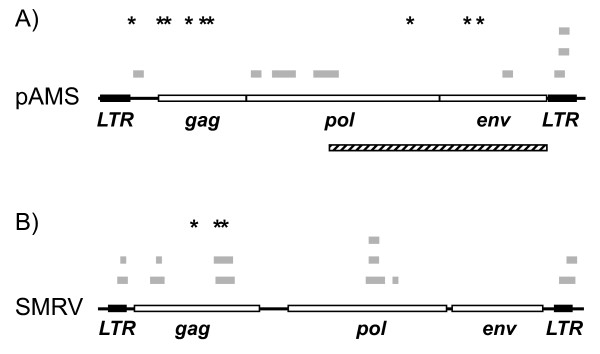
**Mapping of mass spectrometry hits and PAN-PCR products to retroviral genomes**. Asterisks mark the position of peptides identified by mass spectrometry to be derived from pAMS-MLV (A) or SMRV (B), while grey bars indicate genomic regions highly homologous to PAN-PCR products. The hatched box (A) represents proviral sequences of pAMS with high sequence similarity to amphotropic MLV.

Results from PAN-PCR using supernatants of 293T cell cultures confirmed our assumption of retroviral contaminations (Fig 2). While there were no viral sequences detected in the DNA preparation, we found 12 sequences in the RNA preparation that showed high similarity scores (97-100%) to five different regions within either the gag and pol gene or the LTR region of squirrel monkey retrovirus (SMRV, Fig. 2A) by analyses on nucleotide level. Additionally we isolated seven clones that bore six different sequences with high similarity scores of 97-100% to murine leukemia virus strains (MuLV) in a nucleotide BLAST search. These sequences were located within the LTR and the gag, pol and env genes. (Fig. 2B).

To get detailed sequence information for classification of the contaminants and to clarify, if the murine contaminant virus is related or identical to the recently discovered xenotropic MuLV related retrovirus (XMRV) [6] we sequenced the regions of both viruses between the most outwards located subgenomic fragments derived from PAN-PCR. Therefore these regions were amplified by PCR using genomic DNA from infected cells and the primers SMRV-1s (GTTGGGAACCCAGGCTAAGCTG) and SMRV-8057a (GTAGGAGGGGAACCGGCTAC) for SMRV and T3R03s (AGGGGATTTATTGGATACACG), T3R32a (CATCGTGACCTGGGAAGC) for the murine retrovirus. PCR products were sequenced directly by primer walking and resulting proviral genomic sequences were analyzed by a BLASTn search.

The resulting sequence of the simian retrovirus (7.968 kbp) confirmed our preliminary identification as squirrel monkey retrovirus [GenBank: M23385.1] with an overall sequence identity of 98,5%. In contrast the 7.4 kbp sequence of the murine retrovirus was neither identical to one of the murine leukemia viruses nor to XMLV but showed an overall similarity score of 99% to pAMS [GenBank: AF010170], a plasmid carrying the proviral sequence of a recombinant hybrid virus. This construct was engineered in the 1980s and is composed of sequences from Moloney murine leukemia virus (MoMLV) and amphotropic mouse leukemia virus clone 4070A [7,8]. In the current GenBank entry it is described as *"... reference retrovirus for FDA validation of retrovirus vectors used for human gene therapy...". *Neither the hybrid virus itself nor the plasmid pAMS were ever used in our laboratory. Due to the high identity of nucleotide sequence and the fact that the structure of MoMLV and amphotropic leukemia virus related segments is 100% identical to that of pAMS (data not shown) we can exclude that the contaminant virus is a natural recombinant of MoMLV and amphotropic leukemia virus.

In order to confirm our data from PAN-PCR we applied mass spectrometry to supernatants of the 293T cells, suspected to be contaminated. This method was chosen as an additional option - besides PAN-PCR - which allows for identification of contaminants without prior knowledge or assumptions about the agent in question. Therefore, pelleted material from supernatants of transfected and untransfected 293T cells was separated by SDS-PAGE and digested with trypsin. For protein identification peptides were analyzed by nanoLC-ESI-MSMS and uninterpreted ESI-MS/MS-spectra were correlated with the NCBI-protein sequence database applying the SEQUEST™ algorithm [5]. Besides peptides similar to human gene products, we found peptides derived from squirrel monkey retrovirus (SMRV) and murine leukemia virus (MuLV). Additionally there was one hit with the peptide sequence KAADTESGPSSGRT to '*Pol [synthetic construct]*' [GenBank: gi|2281588] corresponding to the integrase of the hybrid amphotropic/Moloney murine leukemia virus (Tables 1 and 2).

**Table 1:** Results of mass spectrometry (nano-LC-ESI-MSMS) - Concentrated and purified supernatant from transfected 293T cells.

Accession number	Descriptive Name
gi|773422	gag protein [SMRV]
gi|40796131	pp12 [Murine leukemia virus] gag
gi|9626959	Pr65 [Murine leukemia virus] gag
gi|4691418	heat shock protein 72 [Homo sapiens]
gi|74589	gag polyprotein [SMRV-H]
gi|9626961	Pr180 [Murine leukemia virus] gag/pol
gi|1045516	gPr80 envelope protein [Murine leukemia virus]
gi|2281588	pol [synthetic product]
gi|30583573	programmed cell death 6 interacting protein [Homo sapiens]

**Table 2:** Results of mass spectrometry (nano-LC-ESI-MSMS) -Concentrated and purified supernatant from untransfected 293T cells.

Accession number	Descriptive Name
gi|773422	gag protein [SMRV]
gi|40796131	pp12 [Murine leukemia virus] gag
gi|18338742	gPr80 glycosylated gag polyprotein [Moloney murine leukemia virus]
gi|42543698	Chain A, The Crystal Structure Of The Human Hsp70 Atpase Domain
gi|1065227	Heat Shock Cognate 70kd Protein (44kd Atpase N-Terminal Fragment)
gi|74589	gag polyprotein [SMRV-H]
gi|9626961	Pr180 [Murine leukemia virus] gag/pol
gi|2281588	pol [synthetic product]
gi|30583573	programmed cell death 6 interacting protein [Homo sapiens]

For quantification of viral genomes from supernatants of cell culture and proviral genomes within cellular DNA we established SybrGreen based Real-Time PCRs (and RT-PCRs). Cellular DNA (from >10^5 ^cells) and viral RNA from supernatants (200 μl) were both purified using the DNA Blood Mini Kit (Qiagen) as recommended by the manufacturer. RNA was treated with RNase-free DNase (Ambion) prior to RT-PCR. Primers were chosen to match one clone derived from PAN-PCR for each virus respectively. For SMRV we used the primers T3R34s (CTGCCCTGTATCATCTGAACC) and T3R34a (CTCCCCTGACATTCAACGC) amplifying the gag gene of the viral genome whereas for the murine hybrid retrovirus primers T3R27s2 (CAGGGAGAACATGGTAATAGGA) and T3R27a2 (ACGACCTCTCCAAAGTATCCA) were used to amplify a region within the env gene. As standards for PCR we used 1 μl of the corresponding plasmid quantified by photometry and diluted to suitable concentrations in water containing herring sperm DNA as carrier/noise DNA (100 ng/μl). Reaction was done using the QuantiTect SybrGreen PCR Kit (Qiagen) in a total volume of 20 μl containing 5 μl purified DNA and 5 pmol of each primer. RNA standards for RT-PCRs were *in vitro *transcripts from the corresponding plasmids. RT-PCR was performed using the QuantiTect SybrGreen RT-PCR Kit (Qiagen) in a total volume of 20 μl with 5 μl purified RNA and 5 pmol of each primer. All quantitative PCRs were performed on a Rotor-Gene 3000 (Corbett Research). After an initial temperature step of 95°C for 15 min 40 cycles of 95°C for 10 s, 55°C for 30s, 72°C for 30 s and acquiring SybrGreen fluorescence at 81°C were performed followed by a standard melting curve analysis of products. In case of RT-PCR a reverse transcription step of 20 min at 50°C was added before cycling.

Quantitative RT-PCRs could confirm high loads in the supernatant of infected 293T cells of up to 10^12 ^RNA copies/ml for the recombinant MLV virus and up to 10^11 ^RNA copies/ml for SMRV. Although these values are rather high, they are not unexpected. Viral RNA copies do not reflect the infectious titer due to a large excess of non-infectious particles. In lentiviral RNA packaging studies we also observed that the RNA copy numbers detected in the supernatants are approximately 1000-fold higher than the infectious titer [9]. Consistently, Münch et al., could increase the titer of HIV by up to 5 orders of magnitude by adding the SEVI peptide, indicating that a vast excess of particles are not detected by conventional titration of infectious virus without SEVI [10]. In addition, viral RNAs released from dying cells independent of budding particles could also contribute to the RNA copies detected in the supernatant of infected cells.

To get further insight into the degree of contamination we quantified proviral genomes within purified cellular DNA from various currently cultured cell lines as well as nitrogen-frozen cell stocks in our laboratory (Table 3). Both viruses showed a broad range of permissiveness for cell lines from multiple species and a first contamination event dated back to the years 2002 (SMRV) and 2003 (hybrid amphotropic/Moloney murine leukemia virus). For both viruses highest infection scores were found for 293T cells, where the mean copy number of proviral genomes per cell reached values of up to 208 for SMRV and 2874 for the pAMS related virus (Table 3). Proviral genomic copy numbers per cell were calculated by the ratio or proviral genomic copy numbers and the DNA content measured by spectrophotometry. To verify the accuracy of determination of cell numbers, standardization by single copy gene PCR for myostatine and quantification of cellular genomic DNA by QBit Kit (Invitrogen) were also performed. Each of the methods used gave comparable results with a maximum deviation of 2.2-fold.

**Table 3:** Proviral load in contaminated cell lines.

	Proviral DNA copies/cell	
	
Cell line	Hybrid amphotropic/Moloney murine leukemia virus	Squirrel monkey retrovirus
CHO	0.0007	0.00002
A549	0.0004	26
Cos 7	0.0005	0.26
HEp2	0.13	0.001
L929	1.44	0.002
Vero E6	3.84	12
3T6	4.58	0.00001
HeLa	5.74	115
293A	1622	20
T-Rex 293	2596	8.11
293T	2874	208

Viral DNA copy numbers were determined by quantitative PCR and are given as the maximum proviral DNA copies/cell detected.

It has to be mentioned that each of both viruses was found in at least one aliquot of all cell lines tested. Thus, we cannot report a single cell line which is not permissive for one of both viruses. Furthermore, experimental infections of retrovirus-negative aliquots of selected cell lines showed that both viruses are highly infectious and propagate to high viral loads as determined by viral RNA copy numbers in the supernatants and proviral genome copies of extracted cellular DNA (data not shown). In the early stage of infection even some cytopathic effects (CPE) could be observed. In contrast, no CPE was seen in persistently infected cultures possibly due to adaptation of cells to the retroviral infection (data not shown). To evaluate a potential contamination of cell lines from tissue culture respositories, we also directly performed PCR analyses of a frozen 293T cell stock obtained from ECACC. Neither proviral DNA of SMRV nor chimeric MLV could be detected.

In summary, there have been numerous publications about retroviral contaminations like recent reports of ecotropic murine leukemia virus in various cell lines [11,12]. The most frequent retrovirus found in this context is squirrel monkey retrovirus (SMRV) [13-16]. One study even reported the detection of SMRV related sequences in commercial interferon preparations in 1998 [17]. Although the sequences were found only as DNA and therefore rather derived from cellular DNA carrying proviral genomes than viral particles, it clearly demonstrated the contamination of the interferon producing cell line with SMRV. Germany's Central Commission of Biosafety (ZKBS) recently reported that SMRV was detectable in 128 samples of 4279 cell cultures from different laboratories throughout the country [18].

The present report extents these studies by identifying for the first time a presumably synthetic chimeric retrovirus as a contaminant. This gene-modified organism seems to have replicated and spread intensely in a broad set of cell lines for several years without being noticed. This hybrid amphotropic/Moloney murine leukemia virus was engineered in the 1980s [7,8] and neither the virus itself nor the plasmid (pAMS) containing its proviral genome were ever used in our laboratory. Although the precise source for the contamination could not be traced back, sharing cell lines with other laboratories seems the most likely explanation. A frozen aliquot of 293T cells (HEK 293tsA201), which we obtained from ECACC, was not contaminated. While SMRV contaminations were detected in different laboratories, testing of three other laboratories' cell lines did not reveal contaminations with the hybrid amphotropic/Moloney murine leukemia virus (data not shown).

This study also shows that both PAN-PCR and mass spectrometry are powerful tools to identify viral contaminations without prior knowledge or assumptions about the viruses in question. Therefore, both methods might be useful in routine controls for viral contaminations of cell cultures.

## Competing interests

The authors declare that they have no competing interests.

## Authors' contributions

AS, EPP, KU and TG wrote the manuscript. EPP performed electron microscopy. EPP and RD were responsible for interpretation of electron micrographs. SB was involved in vesicle production and purification, SL performed mass spectrometry. KS and HEM provided advice for the mass spectrometry analyses. All other experiments and study design were done by AS, KU and TG.
